# Neural substrate of quality of life in patients with schizophrenia: a magnetisation transfer imaging study

**DOI:** 10.1038/srep17650

**Published:** 2015-12-03

**Authors:** Faget-Agius Catherine, Laurent Boyer, Wirsich Jonathan, Ranjeva Jean-Philippe, Richieri Raphaelle, Soulier Elisabeth, Confort-Gouny Sylviane, Auquier Pascal, Guye Maxime, Lançon Christophe

**Affiliations:** 1 Aix-Marseille University, EA 3279, Public Health: Chronic Diseases and Quality of Life, School of Medicine, 13005 Marseille, France; 2 Department of Psychiatry, Conception University Hospital, 13009 Marseille, France; 3 Department of Public Health, Timone University Hospital, Assistance Publique - Hôpitaux de Marseille, 13005 Marseille, France; 4 Aix Marseille University, Centre de Résonance Magnétique Biologique et Médicale (CRMBM) UMR CNRS 7339, Medical School of Marseille, France; 5 Centre d’Exploration Métabolique par Résonance Magnétique (CEMEREM), Medical Imaging Department, Timone University Hospital, AP-HM, Marseille, France

## Abstract

The aim of this study was to investigate the neural substrate underlying quality of life (QoL) and to demonstrate the microstructural abnormalities associated with impaired QoL in a large sample of patients with schizophrenia, using magnetisation transfer imaging. A total of 81 right-handed men with a diagnosis of schizophrenia and 25 age- and sex-similar healthy controls were included and underwent a 3T MRI with magnetization transfer ratio (MTR) to detect microstructural abnormalities. Compared with healthy controls, patients with schizophrenia had grey matter (GM) decreased MTR values in the temporal lobe (BA21, BA37 and BA38), the bilateral insula, the occipital lobe (BA17, BA18 and BA19) and the cerebellum. Patients with impaired QoL had lower GM MTR values relative to patients with preserved QoL in the bilateral temporal pole (BA38), the bilateral insula, the secondary visual cortex (BA18), the vermis and the cerebellum. Significant correlations between MTR values and QoL scores (p < 0.005) were observed in the GM of patients in the right temporal pole (BA38), the bilateral insula, the vermis and the right cerebellum. Our study shows that QoL impairment in patients with schizophrenia is related to the microstructural changes in an extensive network, suggesting that QoL is a bio-psychosocial marker.

Schizophrenia is a chronic, severe, and disabling psychiatric disorder that affects approximately 0.7% to 1% of the general population^1^. Although reducing the severity of symptoms is an important goal for treating patients with schizophrenia, it is well recognised that reducing the symptoms does not entail managing all of the facets that patients consider to be important in their life^2^. The quality of life (QoL) measurements are considered to be increasingly important with regard to evaluating disease progression, treatment and the management of care for patients with schizophrenia^3^. In particular, QoL has been reported to be an independent predictor for long-term symptomatic remission, functional recovery and disability^4^,^5^.

Despite the acknowledged need to consider QoL issues in clinical practice and research, QoL measurement has not been routinely implemented^6^. Among the various reasons for this lack of implementation discussed in the literature^7^,^8^, one concern expressed by clinicians is that they did not feel comfortable with the concept of QoL, or interpreting and including QoL data into medical strategies^9^,^10^. Because QoL is construed historically and traditionally as a psychosocial subjective construct^6^,^11^, many clinicians do not understand the connection between QoL in terms of psychosocial data and the biomedical aspects of patient care. Therefore, a new understanding of the QoL concept provided by building bridges between QoL and neural entities^6^ is necessary to enhance the implementation of QoL data in clinical practice^12^.

Several studies have investigated the neural substrate of QoL in patients with schizophrenia^13^,^14^,^15^. Thus far, only one structural imaging study explored the volumetric abnormalities associated with impaired QoL, using voxel-based morphometry, and revealed grey matter (GM) reductions in the right dorsolateral prefrontal cortex, the left superior frontal sulcus, the left parahippocampal gyrus and the left inferior temporal gyrus^13^. However, recent magnetic resonance imaging (MRI)-based analytical approaches, more sensitive to microstructural changes, have not yet been applied to study the neurobiological substrate of QoL in patients with schizophrenia. In particular, the magnetisation transfer imaging (MTI) has the potential for providing more neuropathological information *in vivo* to subtle or early neuropathological changes than volumetric MRI^16^,^17^. The MT ratio (MTR) provided by MTI allows the accurate estimates of structural abnormalities in the normal-appearing brain tissue, as well as in white matter (WM) and GM^16^,^18^. Moreover, the MTI is a valuable brain-imaging tool that can be used in various psychiatric disorders, such as schizophrenia^16^,^19^,^20^.

The aim of the present study was to investigate the neural substrate underlying QoL by quantifying subtle microstructural abnormalities associated with impaired QoL in a large sample of patients with schizophrenia, using MTI.

## Methods

### Participants

This study was conducted in the Center for Mental Health and Addiction at Conception University Hospital and in the Neuroimaging Department (CEMEREM) at Timone University Hospital (Marseille, France) from April 2011 to December 2013.

Eighty-one right-handed consecutive patients (mean age = 30.0 years, SD = 7.5) with a diagnosis of schizophrenia were included in the study. The inclusion criteria were as follows: being male; aged from 18 years to 45 years; having a diagnosis of schizophrenia according to the Diagnostic and Statistical Manual of Mental Disorders, 4th ed. (DSM-IV-TR) criteria^21^, confirmed by the Structural Clinical Interview (SCID) for DSM-IV-TR^22^, speaking French as the native language; providing informed consent to participate in the study; having a stable disease (no need for hospitalisation at the time of inclusion in the study and no major change in the patient’s condition for 2 months before inclusion in the study)^23^, and being an outpatient. The exclusion criteria were as follows: having a psychiatric diagnosis other than schizophrenia on Axis I of DSM-IV-TR; having mental retardation; and fulfilling any contraindication for MRI examination.

Twenty five healthy right-handed controls (mean age = 29.2 years, SD = 4.8), who were age- and gender-similar to the patients, were included in the study. The controls were free from psychiatric disease on the basis of the SCID^22^ and had no history of neurological or psychiatric disease and no first-degree relatives with psychotic episodes.

The local ethics committee approved the investigations on patients (clinicalTrials.gov identifier: NCT01295411; Comité de Protection des Personnes Sud-Méditerranée V: 2010-A01421-38; AFSSAPS : 11.003) and on controls (Local Ethics Committee for human experimentation, La Timone University Hospital, Marseille, France). Our research was conducted in accordance with the Declaration of Helsinki and French good clinical practices^24^,^25^. In particular, both patients and controls received an explanation of the study and gave their written, informed consent after a standardised and structured clinical interview.

### Data collection

On the same day the MRI was performed, each patient completed the QoL questionnaire and received a standardised psychiatric assessment performed by a psychiatrist using face-to-face interview, clinical examination, and standardised tools.

The following data were collected:

1. QoL measurement: QoL was assessed using the S-QoL 18 questionnaire, which is a self-administered, multidimensional instrument developed and validated for specifically assessing QoL in patients with schizophrenia^26^,^27^,^28^. Because the accuracy and completeness of answers in QoL questionnaires may depend on the questionnaires’ difficulty and length^27^, we have chosen the S-QoL 18, which presents several important properties: the S-QoL 18 is a well-validated questionnaire based exclusively on the patients’ perspectives, ensuring a more appropriate content than the questionnaires based on experts’ determinations^29^. The items of the S-QoL 18 refer to the present time with a one response option, which may be easier for individuals with schizophrenia to understand. Finally, because of its short format and because of the difficulties in concentration and perception characteristic of patients with deficit syndrome or thought disorders, the S-QoL 18 appears to be better adapted to the populations with schizophrenia^27^,^30^. A global score (the index) is calculated whereby the range is from 0, which indicates the lowest QoL, to 100, which indicates the highest QoL.
2. Socio-demographic information: Gender, age, and educational level (primary school or middle school vs. high school) were documented.
3. Clinical characteristics: Duration of disease (defined as time from first contact with mental services) and symptom severity were rated using the Positive and Negative Syndrome Scale (PANSS)^31^. The three subscales of the PANSS (positive, negative, and general psychopathology) are divided into five factors from which hostility-excitation, cognitive and depression factors are of specific interest in our study because of their potential link to QoL^32^.
4. Drug information: Antipsychotic medication (first-generation antipsychotics, FGAs; second-generation antipsychotics, SGAs), chlorpromazine equivalent daily dose, and medications (antidepressants, anxiolytics, and hypnotics) were documented.

### MRI exploration

All of the participants (patients and healthy controls) were examined using an identical MR protocol. All data were obtained using a 3T Magnetom Verio MR Scanner (Siemens, Erlangen, Germany) equipped with a 12-channel head coil. The MR imaging protocol included localiser scout imaging, sagittal 3D FLAIR (fluid attenuated inversion recovery) sequence (TI/TE/TR, 1800 ms/395 ms/5000 ms; 160 contiguous slices; isotropic voxel size, 1 × 1 × 1 mm^3^; bandwidth, 781 Hz/pixel) and sagittal 3D MPRAGE (Magnetization Prepared Gradient Echo) T1-weighted images (TI/TE/TR, 900 ms/2.92 ms/1900 ms; flip angle, 9°; 176 contiguous slices; isotropic voxel size, 1 × 1 × 1 mm^3^; bandwidth, 200 Hz/pixel), and transverse proton, density-weighted, spoiled gradient-echo sequences (750/4.5 ms [TR/TE], 44 contiguous slices; 3-mm section thickness; 30° flip angle; 320 mm FOV, 256 × 256 matrix) performed without and with magnetisation transfer (MT) saturation (1.5-kHz off-water resonance, pulse duration 500 μ).

### Image processing

MTR maps were calculated using a semi-automated method on a voxel-by-voxel basis, according to the following equation: MTR (%) = (*M*_0_ – *M*_mt_)/*M*_0_, where *M*_0_ and *M*_mt_ are the images obtained without and with MT saturation pulse, respectively. The MTR maps were co-registered onto the corresponding T_2_-weighted images of each subject. The MTR maps were spatially normalised into the Montreal Neurologic Institute (MNI) space using the T_1_ anatomic template provided in the SPM8 software (Wellcome Institute, London, UK). After segmentation of the normalised MTR maps using voxel intensities and prior knowledge procedures (SPM8), three maps representing fractions of GM, WM, and CSF were obtained. Pixels with a percentage of tissue (GM + WM) more than 90% were used to mask the normalised MTR map. After normalisation, brain tissue MTR maps were smoothed using a 6-mm Gaussian filter.

### Statistical mapping analysis

First, the microstructural abnormalities of patients with schizophrenia were determined on a voxel-by-voxel basis by comparing the MTR maps of 81 patients with 25 healthy controls using Student’s *t* test (*P* < 0.005; FDR was corrected at a cluster level of *P* < 0.05). The clusters were located on a Talairach atlas^33^ after MNI coordinates were transformed into Talairach coordinates, using a non-linear transform^34^ to infer their architectonic locations in GM (Brodmann Area, BA).

Second, the patients were grouped into preserved and impaired QoL groups, using the population norm of the S-QoL 18 index as the cut-off 26. The patients’ characteristics were compared across both groups using Student’s *t* test or the Mann-Whitney *U* test for continuous variables and a chi-squared test or Fisher’s exact test for frequencies. One-way analysis of variance (ANOVA) and Bonferroni’s post hoc comparison were performed to assess significant differences in MTR maps between patients with impaired QoL, patients with preserved QoL and healthy controls (*P* < 0.005; extent threshold k = 20; FDR corrected at cluster level, *P* < 0.05). Age, PANSS score, disease duration and chlorpromazine equivalent daily dose were considered to be confounding variables.

Finally, correlations were computed between MTR values and S-QoL 18 index scores using a multiple regression model, considering age, disease duration, chlorpromazine equivalent daily dose and PANSS score to be confounding variables (*P* < 0.005; k = 20). The raw data from the surviving clusters were extracted and the correlations from the S-QoL 18 index were confirmed using Spearman rank correlation tests.

## Results

### Clinical and MRI characteristics of the entire group of patients

Table 1 shows clinical characteristics for the 81 patients included in the present study. There was no patient with schizoaffective disorder.

**Figure 1:**
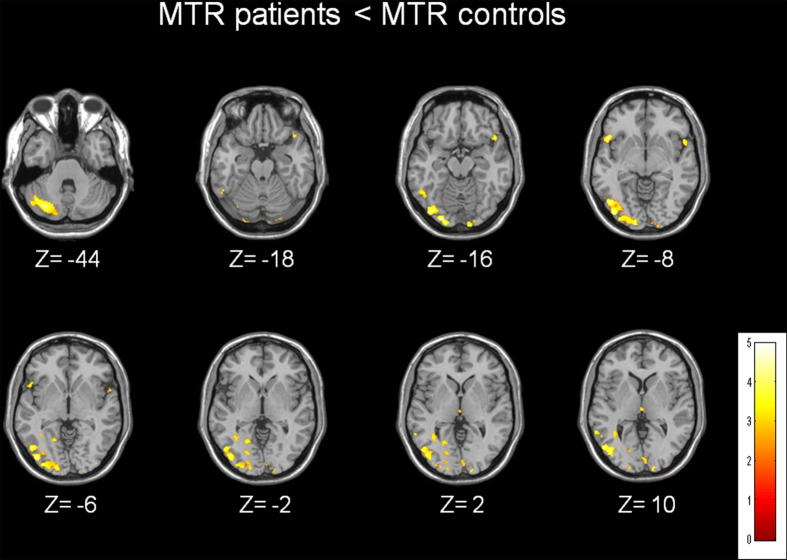
Brain tissue MTR maps of patients compared to controls. Brain regions with GM decrease in MTR values for patients with schizophrenia compared to controls.

Compared to the healthy controls, the entire group of patients had low GM MTR values in the right middle temporal gyrus (BA21), the left temporal pole (BA38), the bilateral insula, the right fusiform gyrus (BA37), the right cuneus (BA17), the right secondary visual cortex (BA18), the right associative visual cortex (BA19) and the right cerebellum. Compared to the control group, the patients exhibited no increase in MTR values in WM and GM and no decrease in the WM (Fig. 1).

### Clinical and MRI differences of patients according to QoL levels

Among the 81 patients with schizophrenia, 44 (54.3%) had a preserved QoL level (Table 1).

**Figure 2:**
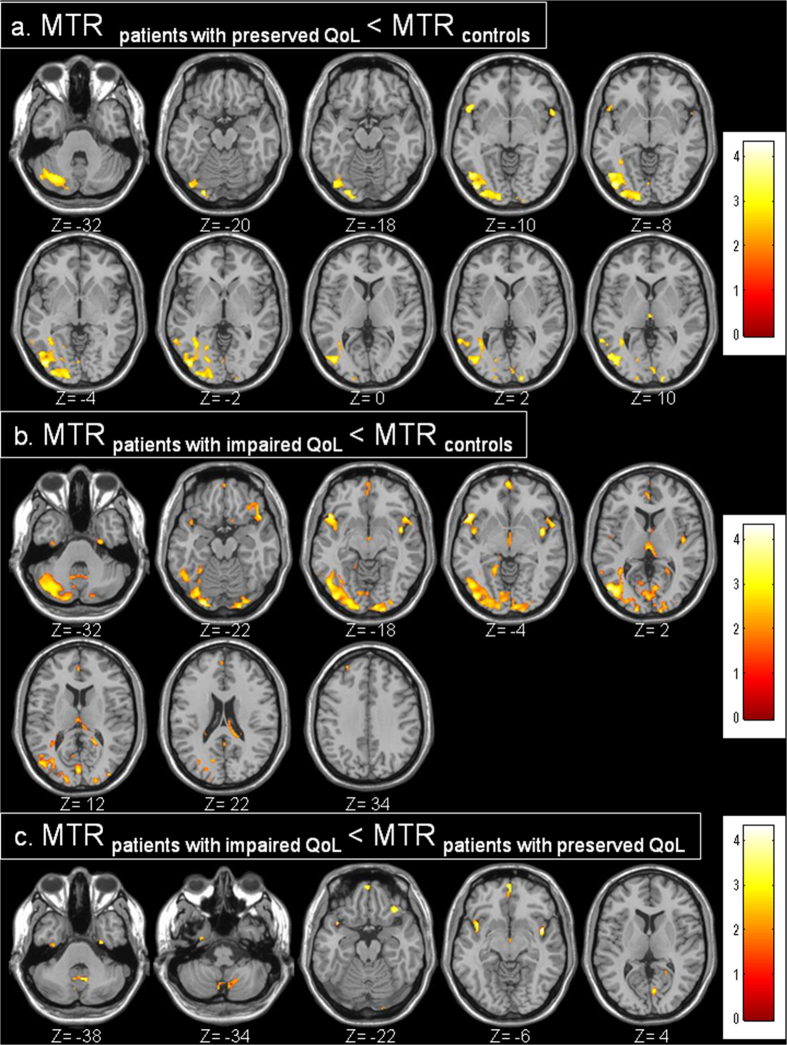
Brain tissue MTR maps of patients with preserved QoL compared to controls (a), of patients with impaired QoL compared to controls (b), and of patients with impaired QoL compared to patients with preserved QoL (c).

Compared to healthy controls, the patients with preserved QoL levels had significant low GM MTR values in the right middle temporal gyrus (BA21), the bilateral insula, the fusiform gyrus (BA37), the right cuneus (BA17), the right secondary visual cortex (BA18), the right associative visual cortex (BA19) and the right cerebellum (Fig. 2a). Compared to healthy controls, the patients with impaired QoL levels had low GM MTR values in the right dorsolateral prefrontal cortex (BA9), the right middle temporal gyrus (BA21), the left superior temporal gyrus (BA22), the bilateral temporal pole (BA38), the bilateral insula, the left dorsal posterior cingulate (BA31), the right inferior temporal gyrus (BA37), the right cuneus (BA17), the bilateral secondary visual cortex (BA18) and the right cerebellum (Fig. 2b).

Compared to patients with preserved QoL levels, the patients with impaired QoL levels had lower MTR values in the GM of the bilateral temporal pole (BA38), the bilateral insula, the left secondary visual cortex (BA18), the vermis and the right cerebellum (Fig. 2c). No other significant difference was found between impaired and non-impaired patients.

No differences between the groups were observed in WM.

### Correlation between MTR values and S-QoL 18 index scores

**Figure 3:**
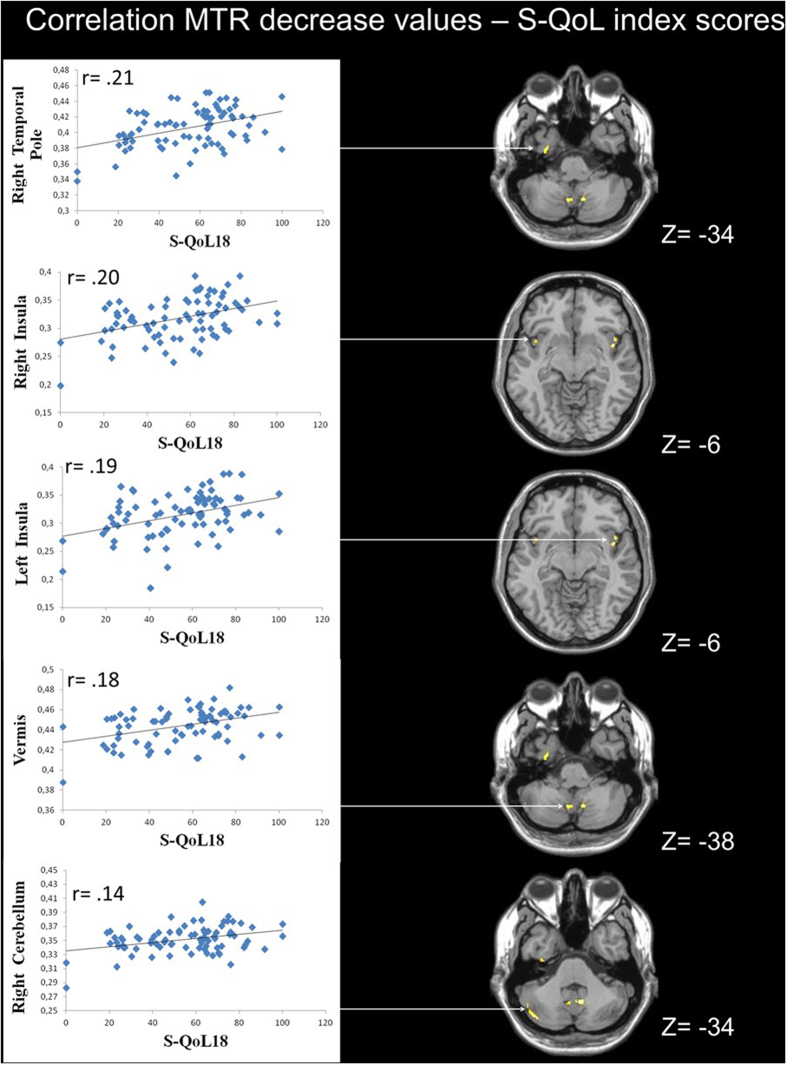
Correlation between MTR values and S-QoL 18 index scores. A low MTR value in the right cerebellum, vermis, temporal pole and bilateral insula was correlated with the S-Qol 18 index scores in patients with schizophrenia.

Significant correlations between MTR values and S-QoL 18 index scores (*p* < 0.005) were observed in the GM of patients (n = 81) within the right temporal pole (BA38) (r = 0.21; *P* < 0.0001), the left insula (r = 0.19; *P* < 0.0005), the right insula (r = 0.20; *P* < 0.0005), the vermis (r = 0.18; *P* < 0.0005) and the right cerebellum (r = 0.14; *P* < 0.005) (Fig. 3).

## Discussion

The present magnetisation transfer imaging study investigates the neural substrate underlying QoL in patients with schizophrenia. Our study first reports significant decreases in GM MTR values that reflect schizophrenia-related microstructural changes compared to healthy controls, confirming the existence of GM microstructural alterations in patients with schizophrenia^35^,^36^. Above all, our study reveals that independent of the usual clinical indicators, the GM MTR decreases were more important in patients with impaired QoL. Our findings, controlled for age, disease duration, symptomatology and medications, obtained by comparing two groups of patients discriminated only by the level of QoL and secondarily confirmed using a correlation analysis in the entire group of patients, highlight the involvement of the temporal pole, the insula and the cerebellum.

Our initial findings report a pattern of GM MTR value decreases in patients with chronic schizophrenia compared to healthy controls, i.e., the temporal lobe (BA21, BA37 and BA38), the bilateral insula, the occipital lobe (BA17, BA18 and BA19) and the cerebellum. These findings using MTI, a sensitive method for evaluating demyelination and axonal loss with a better spatial resolution and less geometric distortions than diffusion tensor imaging, are consistent with previous morphometric studies using region of interest or whole-brain voxel-based morphometry analyses in first-episode and chronic schizophrenia that have reported the existence of GM loss in multiple brain structures^36^. Decreased GM volume in the temporal regions is the most common finding in the comparative studies of patients with schizophrenia and healthy controls^37^,^38^. To the extent that the symptoms can be localised, the temporal region abnormalities have been linked to auditory hallucinations, thought disorder, and memory dysfunction^39^,^40^. GM microstructure impairment within the insula, usually bilaterally, as observed in our study, is also consistent with GM decreases commonly observed in schizophrenia^41^, associated with psychotic symptoms^42^ and cognitive impairments^43^. Cerebellar atrophy^44^, particularly in the vermis^45^, has also been described, affecting cognitive and emotional processing^46^. Finally, although less implicated in the pathophysiology of schizophrenia, a decreased GM volume in the occipital lobe has been detected^47^ supporting dysfunctions of cognitive or visual processing^48^. Altogether, our results demonstrate MTR GM decreases in crucial brain regions that involve several functions that are altered in schizophrenia. Unlike previous studies, our results failed to identify WM MTR value decreases^17^,^49^. This discrepancy may be explained by the heterogeneity of chronic schizophrenia population (e.g., duration of illness, medications).

Second, a neural understanding of QoL may address the concerns of healthcare professionals regarding the meaning of this measure, its relevance and its usefulness in clinical practice.

An impaired QoL was associated with more microstructural changes in distributed GM areas that correspond to particular functional networks. These findings implies that QoL is influenced by a neurological background, suggesting that QoL could be considered to be a bio-psychosocial construct rather than only a psychosocial construct^6^. The brain areas involved in the QoL levels (i.e., the temporal pole, the insula and the cerebellum) provide information concerning what is measured using a QoL questionnaire. Our findings suggest that QoL is closely linked to a functional brain network regulating emotional behaviour. On the one hand, the insula integrates perceptions, emotions and thoughts into a subjective word^50^ and has been involved in linking emotion signals with other sensory information. Interestingly, a dopaminergic abnormality is likely to be associated with this brain area in patients with schizophrenia^51^ and dopamine has been implicated in the mediation of pleasurable experiences^52^. From a network perspective, insula plays a key role in the salience network and especially in the switching between the default mode network and the central executive network^51^,^53^,^54^,^55^,^56^. The anterior frontal region involved in significant MTR decreased in impaired patients compared to non-impaired could also be part of the salience network. On the other hand, the right temporal pole has been associated with metacognition (i.e., the ability to attribute mental states, in terms of beliefs and goals, to oneself and others)^57^, which plays a central role in the understanding, learning and regulation of emotions^58^. Moreover, neural connections have been reported between this temporal area and the insula^59^. Finally, the cerebellum, and particularly the vermis, is also involved in emotional behaviour^60^. The involvement of all these brain areas is also consistent with a recent neuroimaging studies in fibromyalgia in which QoL was associated with right medial temporal metabolism within the limbic system, which is involved in the affective and emotional domains^61^. Another study found that in schizophrenia, QoL was associated with the superior temporal sulcus involved in metacognition^12^. Considering all of these points, we may hypothesise that QoL provides information regarding the emotional experience and social interactions of individuals.

The persistency of the link between QoL and the microstructural changes in the GM after adjustment for the usual clinical indicators suggests that QoL adds information to the data traditionally collected and used in psychiatry. QoL should not be considered to be a derivative of symptomatology and other “objective” factors (e.g., functioning, socio-economic factors) although moderate links exist^62^,^63^ but rather a unique and relevant perspective of the patient with regard to his health.

Finally, the neural underpinnings of QoL could open interesting perspectives for using QoL as a bio-psychosocial marker in the evolution of schizophrenia. QoL has been reported to be an independent predictor for the long-term prognosis of schizophrenia and other chronic diseases, sometimes more than the severity of symptoms^5^,^64^,^65^. In our study, the association of QoL impairment with microstructural changes in core regions of functional networks involved in emotional and social processing suggests that QoL may precociously capture progressive brain tissue loss involved in the prognosis of schizophrenia^66^,^67^. This hypothesis presents the likelihood that QoL could guide individual therapeutic strategy, especially to identify early those patients with more important progressive brain loss and thus with poorer long-term prognosis. The early detection of these patients may allow proposing more adapted treatment. This hypothesis should be explored and confirmed in future longitudinal studies.

Several limitations of the study should be carefully considered. First, the sample may not be representative of the entire population of patients with schizophrenia. All patients were males, had a moderate illness, were middle-aged and had more than 5 years of disease duration. Additional exploration on more diverse and larger groups of patients is therefore required to corroborate our findings. Second, the relatively small sample size for the control group can be a limitation. However, their characteristics were similar to those of the patients. Third the cross-sectional design of our study precludes any conclusions regarding the directionality of the association between QoL and GM abnormalities in schizophrenia. Future longitudinal studies should specifically explore this issue. Fourth, our findings could have been blurred by the impact produced by the antipsychotic medications for the likely effects on the structure or functioning of the brain. However, because the patient groups were for the most part comparable, the results observed should be interpreted in terms of differences in QoL levels. Last, we chose to explore the neural substrate underlying QoL by focusing on the microstructural abnormalities using MTI. It would be interesting to study the functional neural substrate underlying QoL using resting state functional MRI approach and connectivity analysis.

## Conclusion

Our study shows that QoL impairment in patients with schizophrenia is related to microstructural changes in distributed areas usually participating to functional networks involved in emotional and social interactions processes, suggesting that QoL provides unique information concerning the emotional and social experience of individuals who are not available for traditional assessments. Additionally, this study could present interesting perspectives for using QoL as a bio-psychosocial marker in the evolution of schizophrenia.

## Additional Information

**How to cite this article**: Catherine, F.-A. *et al.* Neural substrate of quality of life in patients with schizophrenia: a magnetisation transfer imaging study. *Sci. Rep.* **5**, 17650; doi: 10.1038/srep17650 (2015).

## Figures and Tables

**Table 1:** Socio-demographic and clinical characteristics of the study sample (n = 81).

Characteristics	Entire Sample (n = 81)	Impaired QoL (n = 37)	Preserved QoL (n = 44)	Impaired vs. Preserved QoL *P*
Age (years), mean (SD)	30.0 (7.5)	30.3 (8.2)	29.7 (6.9)	0.72
Educational level (high school), no. (%)	64 (79.0)	28 (75.7)	36 (81.8)	0.32
Duration of disease (years), mean (SD)	7.2 (4.6)	6.6 (5.1)	7.7 (4.1)	0.28
S-QoL 18 index score, mean (SD)	54.4 (22.0)	34.5 (13.9)	71.2 (10.2)	<0.001
PANSS score, mean (SD)	73.5 (20.8)	76.7 (23.7)	70.8 (17.7)	0.20
*Positive factor*	14.5 (5.8)	15.6 (6.0)	13.6 (5.6)	0.14
*Negative factor*	29.1 (9.6)	33.4 (6.7)	25.6 (10.2)	<0.001
*Hostility-excitation factor*	10.8 (5.5)	11.4 (5.9)	10.3 (5.2)	0.36
*Depressive factor*	6.2 (2.7)	7.0 (2.7)	5.6 (2.6)	0.01
*Cognitive factor*	12.9 (4.5)	13.8 (3.7)	12.1 (5.0)	0.10
Antipsychotics, no.				
*Second-generation antipsychotics*	75	34	41	0.80
*First-generation antipsychotics*	5	2	3	0.66
*No antipsychotic treatment*	1	1	0	NA
*Antidepressants*	17	9	8	0.81
*Anxiolytics*	9	3	6	0.32
*Hypnotics*	5	3	2	0.66
*Chlorpromazine equivalent daily dose, mean (SD)*	431.1 (352.6)	506.4 (392.9)	367.8 (305.1)	0.08

S-QoL 18, Schizophrenia Quality of Life questionnaire; QoL, Quality of Life; PANSS, Positive and Negative Symptoms Scale; SD, standard deviation.
