# Corrigendum: Neural substrate of quality of life in patients with schizophrenia: a magnetisation transfer imaging study

**DOI:** 10.1038/srep21055

**Published:** 2016-02-15

**Authors:** Catherine Faget-Agius, Laurent Boyer, Jonathan Wirsich, Jean-Philippe Ranjeva, Raphaelle Richieri, Elisabeth Soulier, Sylviane Confort-Gouny, Pascal Auquier, Maxime Guye, Christophe Lançon

Scientific Reports 5: Article number: 17650; 10.1038/srep17650 published online: 12032015; updated: 02152016

The original version of this Article contained errors in the spelling of the authors Catherine Faget-Agius, Jonathan Wirsich, Jean-Philippe Ranjeva, Raphaelle Richieri, Elisabeth Soulier, Sylviane Confort-Gouny, Pascal Auquier, Maxime Guye and Christophe Lançon which were incorrectly given as Faget-Agius Catherine, Wirsich Jonathan, Ranjeva Jean-Philippe, Richieri Raphaelle, Soulier Elisabeth, Confort-Gouny Sylviane, Auquier Pascal, Guye Maxime & Lançon Christophe respectively.

In addition, the ‘How to cite this article’ section quoted an incorrect abbreviation for Catherine Faget-Agius.

These errors have now been corrected in the PDF and HTML versions of the Article.

